# The ongoing challenge of vancomycin-resistant *Enterococcus faecium* and *Enterococcus faecalis* in Europe: an epidemiological analysis of bloodstream infections

**DOI:** 10.1080/22221751.2020.1769500

**Published:** 2020-06-04

**Authors:** Olaniyi Ayobami, Niklas Willrich, Annicka Reuss, Tim Eckmanns, Robby Markwart

**Affiliations:** Unit 37: Nosocomial Infections, Surveillance of Antimicrobial Resistance and Consumption, Robert Koch Institute, Berlin, Germany

**Keywords:** Vancomycin-resistant *Enterococcus faecium*, vancomycin-resistant *Enterococcus faecalis*, vancomycin-resistant enterococci, VRE, VREF

## Abstract

Vancomycin-resistant enterococci infections are of great public health significance due to limited therapeutic options. We investigated epidemiological trends and risk factors of vancomycin resistance in enterococci isolates from patients with bloodstream infections in the EU/EEA from 2012 to 2018. Routine vancomycin susceptibility data of clinical *E. faecium* (n = 67,022) and *E. faecalis* (n = 103,112) blood isolates from the European Antimicrobial Resistance Surveillance Network were analysed using descriptive statistics and multivariable regression analyses. In Europe, proportions of vancomycin-resistant *E. faecium* (VREFm) increased from 8.1% (95%CI 6.7–9.7%) in 2012 to 19.0% (95%CI 16.8–21.5%) in 2018. Rising VREFm proportions were observed across all European regions, both genders and all age groups except children and adolescents (1–19 years). Adults (20–59 years) and elderly (≥60 years) had an increased likelihood of VREFm compared to children and adolescents (1–19 years) (OR: 1.99 [95%CI 1.42–2.79, p < 0.001] and OR: 1.56 [95%CI 1.09–2.23, p = 0.014], respectively). Inpatients hospital units, including internal medicine and ICUs, were associated with an increased likelihood of VREFm (OR: 2.29 (95%CI 1.58–3.32, p < 0.001) compared to the emergency department which reflects patients with community origin of *E. faecium* infections. The mean proportion of vancomycin-resistant *E. faecalis* in Europe was found to be low (1.1% [95%CI 0.9–1.4%]). Local and regional authorities should intensify efforts directed at diagnostic and antimicrobial stewardship for vancomycin and all last resort drugs for the management of VREFm, particularly for hospitalized elderly patients.

## Introduction

*Enterococcus spp.* are Gram-positive bacteria that commonly inhabit the intestinal tracts of healthy humans and animals but have the potential to cause invasive infections if the delicate microbiota balance is disrupted [1,2]. They have adapted to colonizing and persisting in a hospital environment, allowing for easy transmission through multiple routes of cross contamination including invasive medical devices [2–4]. *Enterococcus faecium* (*E. faecium*) and *Enterococcus faecalis* (*E. faecalis*) are the most frequently isolated species in nosocomial settings [5]. Globally, both species are commonly associated with hospital outbreaks of bacteremia, urinary tract infections, endocarditis amongst others [5,6]. Such outbreaks not only result in significant economic costs to health systems, they also risk exposing vulnerable patients to potentially fatal infection [3,7,8]. This challenge is compounded by treatment difficulties associated with the development of high-level resistance to several antibiotics that are either intrinsic or acquired through horizontal transfer of plasmids and transposons [7–9]. Vancomycin-resistant *E. faecium* (VREFm) has been identified as the leading multidrug-resistant *Enterococcus spp.* in healthcare environments [3,10,11]. Due to its clinical and public health significance, the World Health Organization (WHO) and the U.S. Centers for Diseases Control and Prevention listed VREFm as a high priority pathogen in urgent need of drug research and development [12,13]. The European Antimicrobial Resistance Surveillance Network (EARS-Net) reported that the mean proportion of vancomycin-resistant *E. faecium* in invasive isolates was 17.3% (95% CI 17–18) in 2018 compared to 10.4% (95% CI 10–11) in 2014 in countries of the European Union and European Economic Area (EU/EEA) [9]. The increasing proportion of VREFm has also been documented at the country level (e.g. Germany, Italy, Slovakia, and Norway) within the EU/EEA [9,14,15]. While human infections with vancomycin-resistant *E. faecalis* have been reported worldwide, *E. faecalis* has remained generally susceptible to vancomycin compared to *E. faecium* in these regions, including Europe [16–22].

Despite the existing evidence, a comprehensive epidemiological picture of invasive vancomycin-resistant enterococci in Europe is lacking. In particular, it has not been systematically assessed how patient characteristics (such as age) and healthcare setting (such as intensive care) are associated with the likelihood of vancomycin resistance in *E. faecium* bloodstream infections*.* Although a general increase of VREFm is reported for Europe, it is not known which patient groups are affected by rising VREFm proportions, which is crucial for the targeted implementation of infection control and prevention programs. To deepen the understanding of the increasingly problematic enterococci infections, this study aimed to analyse epidemiological trends of vancomycin-resistant *E. faecium* and *E. faecalis* and to determine factors that are associated with an increased likelihood of vancomycin resistance in *E. faecium* blood isolates using EARS-Net data from 2012 to 2018.

## Methods

### Study design and the European antimicrobial resistance surveillance database

We conducted a retrospective observational study on *E. faecium* and *E. faecalis* (2012–2018) using data retrieved from the *European Antimicrobial Resistance Surveillance Network* (EARS-Net) database (TESSy). EARS-Net is a network of European surveillance systems that collects routine clinical antimicrobial susceptibility (AST) data on invasive isolates (blood and cerebrospinal fluid (CSF)) from the 27 countries in the European Union as well as Norway, Iceland and the United Kingdom [13]. Detailed information about the methodology of EARS-Net is provided in the EARS-Net surveillance reports and protocols [14]. In EARS-Net, *E. faecium* and *E. faecalis* isolates are classified as sensitive (S), intermediate (I), or resistant (R) to antimicrobial drugs based on the standards used in the participating laboratories, e.g. guidelines of the European Committee on Antimicrobial Susceptibility Testing (EUCAST), Clinical and Laboratory Standards Institute (CLSI) or other national guidelines. In addition to S-I-R data, individual laboratories provide further epidemiological information, such as date of specimen collection, country of origin, specimen type (i.e. blood and CSF), hospital unit (e.g. intensive care unit [ICU] or general units like internal medicine unit), patient gender and patient age.

### Selection of enterococci isolates

In October 2019 we extracted 2012–2018 data for *E. faecium* and *E. faecalis* from the TESSy database with the approval of the European Centre for Disease Prevention and Control. All enterococci isolates were of bloodstream origin. The TESSy database of EARS-Net only includes the first isolate from a given patient in the respective year. To identify unique isolates, we created a composite identifier comprised of the reporting country, unique laboratory identifier, hospital identifier, patient identifier, date of sample collection and the identified pathogen. Isolates with duplicate composite identifiers and more than one AST against the same antibiotic were excluded. In the next steps, we excluded isolates that were not tested against vancomycin, were from outpatients or were not assigned a hospital ID.

### Variables

Patient age was categorized into four age categories (<1, 1–19, 20–59, ≥60 years). Patient gender was classified into female or male. The country of origin of the isolate was grouped into four major regions of Europe (**North:** Denmark, Finland, Iceland, Ireland, Norway, Sweden, United Kingdom; **West:** Austria, Belgium, France, Germany, Luxembourg, Netherlands; **South:** Croatia, Cyprus, Greece, Hungary, Italy, Malta, Portugal, Slovenia, Spain; **East:** Bulgaria, Czech Republic, Estonia, Latvia, Lithuania, Poland, Romania, Slovakia). Hospital unit types were categorized into emergency department, intensive care unit (ICU), internal medicine, surgery, oncology, and other units. In order to investigate the population-weighted proportion of co- and cross-resistance of vancomycin-resistant *E. faecium* isolates to ampicillin, amoxicillin, linezolid, gentamicin and teicoplanin, only isolates that were tested separately against each of ampicillin, amoxicillin, linezolid, gentamicin and teicoplanin, respectively, were selected. Isolates were defined as ampicillin-, amoxicillin-, linezolid-, gentamicin- and teicoplanin-resistant if they were tested “resistant” against these antibiotics.

### Outcomes and statistical analyses

The primary outcome was the population-weighted proportion of vancomycin-resistant *E. faecium* and *E. faecalis* isolates among all *E. faecium* and *E. faecalis* isolates, respectively, expressed in percentage (%) and 95% confidence intervals (95% CI). An isolate was defined as vancomycin-resistant if it was tested resistant or intermediate against vancomycin. Importantly, among *E. faecium* isolates identified as “resistant” with our definition, only 0.7% were tested as intermediate.

The potential association between different variables and vancomycin resistance of *E. faecium* isolates was analysed using univariable and multivariable logistic regression analyses. For univariable analyses, the following predictors for vancomycin resistance were considered: Year of sampling, gender, age group, European region, and hospital unit type. These variables were selected before the analysis based on the availability of data and our prior hypotheses about variables that may be associated with vancomycin resistance in *E. faecium*. We included all variables from the univariable analyses in the model for the multivariable analysis. In order to analyse whether the yearly change of VREFm proportions is associated with a particular patient and healthcare characteristics, four distinct multivariable logistic regression analyses were performed, including the interaction of year of sampling with each of the four aforementioned variables separately. Individual adjusted odds ratios for VREFm time trends for (i) European region, (ii) gender, (iii) age and (iv) care type were extracted from the multivariable regression by using linear combinations of the log-odds of the coefficient for year and the respective interaction coefficient.

All statistical analyses were performed using R version 3.6.1 [23] and the “survey” package (version 3.37) [24]. For all analyses in all strata we accounted for clustering at hospital level and applied country population-based weighting. The population data were obtained from the Eurostat database [25]. Country population weighting was used to ensure that the data from each country contributed proportionally (in relation to its population size) to the calculation of resistance proportions. This was done to minimize bias from significant differences in isolate numbers from various countries.

## Results

### Baseline characteristics of included *E. faecium* blood isolates

The baseline characteristics of the analysed *E. faecium* isolates are outlined in Table 1. A total of 67,022 blood isolates of *E. faecium* from 63,459 patients were collected in 2057 hospitals across Europe from 2012 to 2018. The majority of the isolates originated from elderly patients (median age: 69 years, IQR: 59–78 years). For the isolates with a reported patient gender (*n* = 61,423, 91.6%), the female/male ratio was 0.65. The inpatient hospital units accounted for the vast majority (96.5%) of the isolates, with about a quarter each derived from patients treated in ICUs (25.4%) and internal medicine units (23.2%). 3.5% of *E. faecium* isolates were recorded among patients seen in the emergency department. About two-thirds of the isolates (64%) were from the Northern and Western regions of Europe, which represented 55% of the total population of the 30 countries included in the study.

**Table 1.** Baseline characteristics of blood isolates of *Enterococcus faecium* and *Enterococcus faecalis* in the EU/EEA

	*E. faecium*		*E. faecalis*	
*Number of isolates (%)*	67,022	100	103,112	100
*Year of sampling*				
2012 (*n*, %)	6,852	10.22	10,775	10.45
2013 (*n*, %)	7,892	11.78	12,039	11.68
2014 (*n*, %)	7,831	11.68	12,242	11.87
2015 (*n*, %)	8,688	12.96	13,759	13.34
2016 (*n*, %)	11,567	17.26	16,862	16.35
2017 (*n*, %)	11,549	17.23	18,216	17.67
2018 (*n*, %)	12,643	18.86	19,219	18.64
*European regions*				
North (*n*, %)	17,766	26.51	20,971	20.34
West (*n*, %)	24,972	37.26	39,743	38.54
South (*n*, %)	17,761	26.50	30,723	29.80
East (*n*, %)	6,523	9.73	11,675	11.32
*Gender of patients*				
Female (*n*, %)	24,119	35.99	31,899	30.94
Male (*n*, %)	37,304	55.66	62,132	60.26
NA (*n*, %)	5,599	8.35	9,081	8.81
Sex ratio (f/m)	0.65			0.51
*Age of patients*				
<1 year (*n*, %)	711	1.06	3,505	3.40
1–19 years (*n*, %)	936	1.40	1,570	1.52
20–59 years (*n*, %)	14,521	21.67	18,575	18.01
60+ years (*n*, %)	46,191	68.92	72,242	70.06
NA (*n*, %)	4,663	6.96	7,220	7.00
Age (median, IQR)	69 yrs	59–78 yrs	71 yrs	60–80 yrs
*Hospital unit type*				
Emergency Unit	2,357	3.52	8,218	7.97
Intensive care unit	17,009	25.38	18,773	18.21
Internal medicine	15,545	23.19	27,230	26.41
Surgical unit	7,356	10.98	3,730	3.62
Oncology	5,696	8.50	17,007	16.49
Other (*n*, %)	6,374	9.51	10,545	10.23
NA (*n*, %)	12,685	18.93	17,609	17.08
*Number of hospitals*	2,340	*** ***	2,527	*** ***

Note*:* EU: European Union, EEA: European Economic Area; IQR: Interquartile range; NA: not available; yrs: Years.

### Temporal and spatial trends of VREFm in the Europe

**Figure 1.**
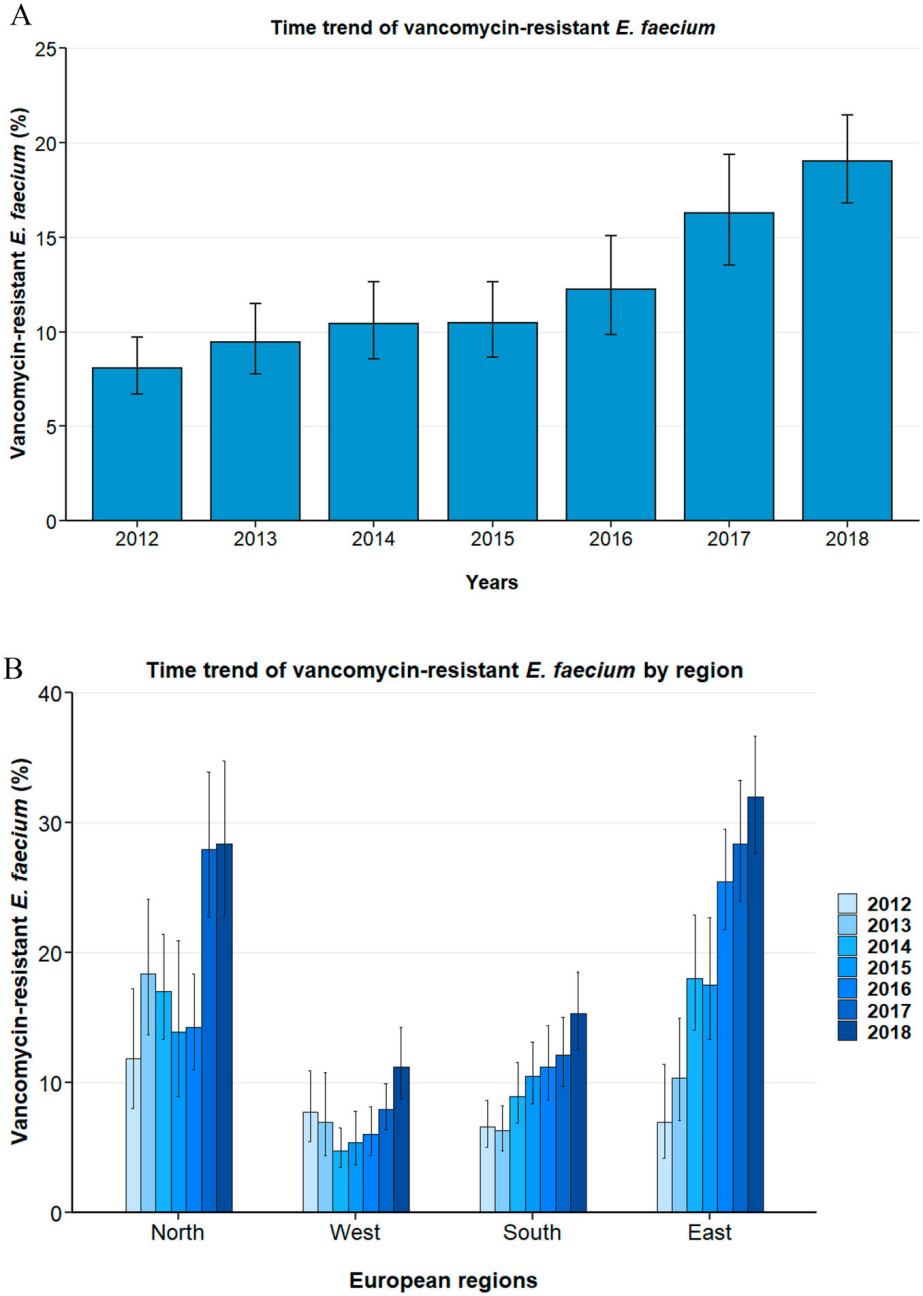
Time trend of vancomycin-resistant *E. faecium* from blood isolates *in the* EU/EEA. Time trend of vancomycin-resistant *Enterococcus faecium* in (A) 30 countries of the European Union, European Economic Area and the United Kingdom, and in (B) major regions within Europe. Vancomycin resistance proportions are expressed as population-weighted mean proportions (%) among all *Enterococcus faecium* blood isolates, with corresponding 95% confidence intervals.

**Additional Figure 1.**
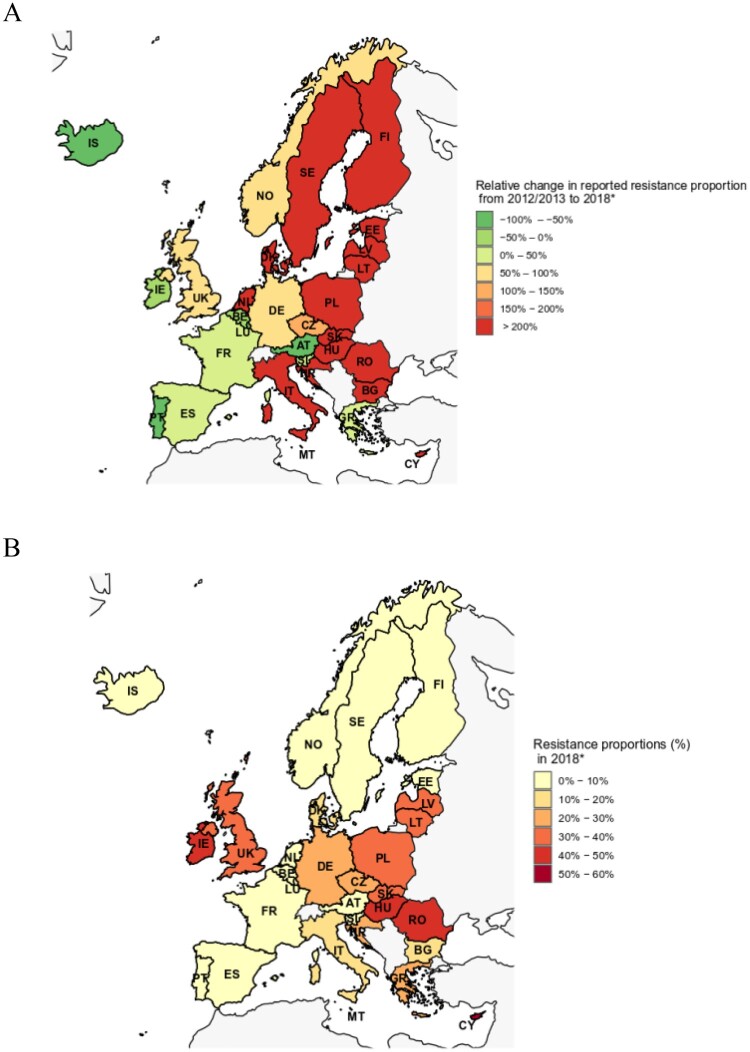
Vancomycin-resistant *E. faecium* blood isolates in the EU/EEA. (A) Relative changes (in %) of proportions of vancomycin-resistant *Enterococcus faecium* (VREFm) among all *E. faecium* blood isolates between 2012/2013 (aggregated) and 2018* in European Union (EU) and Euroepan Economic Area (EEA). (B) Absolute VREFm proportions in 2018 (in %, VREFm isolates among all *Enterococcus faecium* blood isolates) in EU/EEA countries. * Since for Slovenia no data for 2018 were available, 2017 data were used. AT: Austria, BE: Belgium, BG: Bulgaria, CY: Cyprus, CZ: Czech Republic, DE: Germany, DK: Denmark, EE: Estonia, ES: Spain, FI: Finland, FR: France, GR: Greece, HR: Croatia, HU: Hungary, IE: Irleland, IS: Iceland, IT: Italia, LT: Lithunia, LU: Luxembourg, LV: Latvia, MT: Malta, NL: Netherlands, NO: Norway, PL: Poland, PT: Portugal, RO: Romania, SK: Slovakia, SL: Slovenia, UK: United Kingdom.

In the study period 2012 through 2018, the population-weighted mean proportion of VREFm in blood isolates in Europe was 13.0% (95% CI 11.4–14.8%). There was an increase from 8.1% (95% CI 6.7–9.7%) in 2012 to 19.0% (95% CI 16.8–21.5%) in 2018 (Fig. 1A). The yearly increase of VREFm was found to be statistically significant in a multivariable regression analysis (OR: 1.28 [95%CI 1.24–1.33, *p*<0.001]) adjusting for factors that might impact VREFm likelihood, such as European region, hospital unit category, patient age and gender (Table 2). Importantly, between 2012 and 2018, rising trends of VREFm proportions were observed in all European regions and in the majority of countries (Fig. 1B and additional Figure 1A). In the Northern and Eastern regions, VREFm proportions increased from 11.9% (95% CI 8.0–17.3%) and 7.0% (95% CI 4.2–11.4%) in 2012 to 28.4% (95% CI 22.8–34.8%) and 32.0% (95% CI 27.7–36.7%) in 2018, respectively. In Western and Southern regions VREFm proportions rose from 7.8% (95% CI 5.5–10.9%) and 6.6% (95% CI 5.0–8.7%) to 11.2% (95% CI 8.7–14.3%) and 15.3% (95% CI 12.6–18.5%), respectively. Multivariable analysis including interaction between European region and year while adjusting for other variables potentially affecting resistance proportions confirmed the increase of VREFm proportions in all four European regions studied (Table 3). Interestingly, from 2012 to 2018, noticeable regional differences in VREFm proportions were observed in Europe. The population-weighted mean proportion of VREFm was 22.3% (95% CI 19.7–25.2%) and 18.2% (95% CI 15.8–20.8%) in the Eastern and Northern region, respectively, compared to 11.3% (95% CI 9.8–12.9%) and 7.6% (95% CI 6.0–9.6%) in the Southern and Western region, respectively. Univariable and multivariable regression analyses confirmed that *E. faecium* blood isolates from Eastern and Northern Europe were more likely to be vancomycin-resistant than isolates from Southern and Western European regions (Table 2). The geographical differences were also still apparent in 2018. While the Eastern and Northern regions reported VREFm proportions of 32.0% (95%CI 27.7–36.7%) and 28.4% (95%CI 22.8–34.8%), respectively, proportions were 15.3% (95%CI 12.6–18.5%) and 11.2% (95%CI 8.7–14.3%) in the Southern and Western regions, respectively (Fig. 1B and additional Figure 1B).

**Table 2.** Analysis of factors associated with vancomycin resistance in *E. faecium* blood isolates in the EU/EEA.

		Univariable analysis			Multivariable analysis		
		OR	(95% CI)	*p*-value	OR	(95% CI)	*p*-value
*Year of sampling*							
per year		1.21	(1.17–1.25)	<0.001	1.28	(1.24–1.33)	<0.001
*European region*							
North		1	–	–	1	–	–
West		0.37	(0.28–0.50)	<0.001	0.48	(0.33–0.69)	<0.001
South		0.57	(0.46–0.72)	<0.001	0.70	(0.52–0.93)	0.015
East		1.30	(1.03–1.63)	0.028	1.68	(1.26–2.23)	<0.001
*Gender*							
Female		1	–	–	1	–	–
Male		0.87	(0.81–0.95)	<0.001	0.91	(0.84–1.0)	0.041
*Age*							
<1 year		0.63	(0.41–0.97)	0.035	0.67	(0.41–1.10)	0.114
1–19 years		1	–	–	1	–	–
20–59 years		1.54	(1.16–2.06)	0.003	1.99	(1.42–2.79)	<0.001
60+ years		1.17	(0.88–1.54)	0.279	1.56	(1.09–2.23)	0.014
*Hospital unit category*							
Emergency department		1	–	–	1	–	–
Inpatient hospital units		2.29	(1.56–3.37)	<0.001	2.29	(1.58–3.32)	<0.001

Note: 95% CI: 95% confidence interval; OR: Odds ratio.

**Table 3.** Adjusted odds ratios (aOR) for time trends (2012–2018) of VREFm proportions based on four distinct multivariable analyses including interaction of year with region, gender, age or hospital unit category, respectively, additionally to the variables included before (see Table 2).

	aOR	(95% CI)	*p*-value
*1. Time trends by region*			
North	1.45	(1.30–1.61)	<0.001
West	1.20	(1.12–1.29)	<0.001
South	1.29	(1.19–1.39)	<0.001
East	1.32	(1.25–1.39)	<0.001
*2. Time trends by gender*			
Female	1.23	(1.23–1.36)	<0.001
Male	1.27	(1.22–1.33)	<0.001
*3. Time trends by age*			
<1 years	1.53	(1.24–1.89)	<0.001
1–19 years	1.10	(0.96–1.27)	0.177
20–59 years	1.25	(1.18–1.32)	<0.001
60+ years	1.30	(1.24–1.36)	<0.001
*4. Time trends by hospital unit category*			
Emergency department	1.11	(0.97–1.28)	0.121
Inpatient hospital units	1.29	(1.24–1.34)	<0.001

Note: 95% CI: 95% confidence interval; aOR: Adjusted odds ratio.

### Age and gender

**Figure 2.**
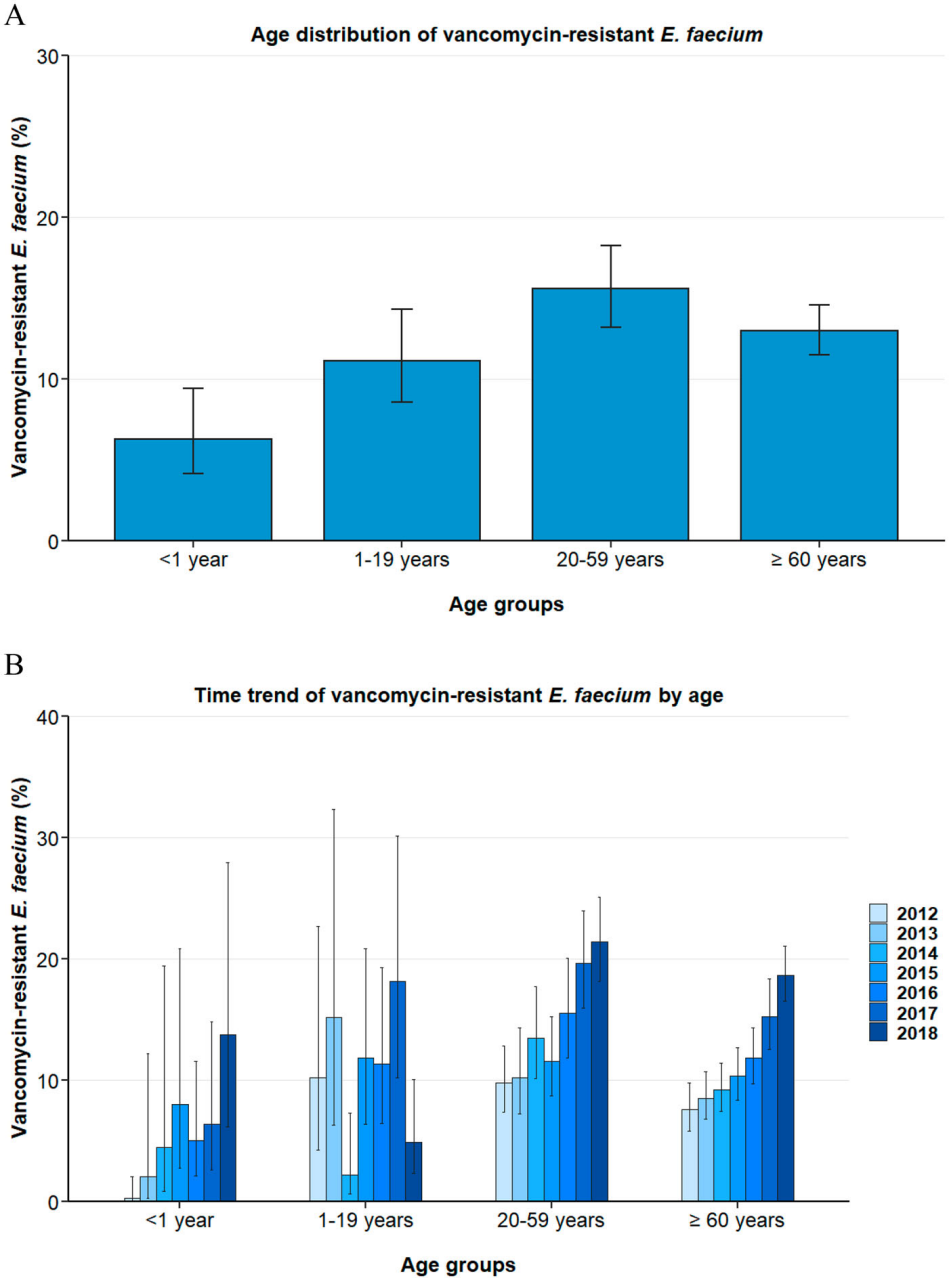
Vancomycin-resistant *E. faecium* from blood isolates stratified into age. (A) Vancomycin-resistant *Enterococcus faecium* stratified into age, expressed as population-weighted mean proportions (%) among all *Enterococcus faecium* blood isolates, with corresponding 95% confidence intervals. (B) Time trends of vancomycin-resistant *Enterococcus faecium* in different age groups.

To determine any underlying influence of the age and gender of hospitalized patients on VREFm proportions, we analysed any association between these demographic variables and VREFm. The data displayed in Fig. 2 show that isolates from infants (<1 year) exhibited lower VREFm proportions (6.3% [95% CI 4.2–9.4%]) than adults (20–59 years; 15.6% [95% CI 13.2–18.3%]) and elderly patients [≥60 years; 13.0% (95%CI 11.5–14.6%) (Fig. 2A)]. In addition, our data suggest that children and adolescents (1–19 years) also showed lower VREFm proportions (11.1% [95% CI 8.6–14.3%]) than the older age groups, which was confirmed by multivariable analysis (Table 2).

Importantly, the increase of VREFm proportions from 2012 to 2018 was found in blood isolates from infants (<1 year, adjusted OR: 1.53 [95 CI 1.24–1.89, *p*<0.001]), adults (20–59 years, adjusted OR: 1.25 [95% CI 1.18–1.32, *p* < 0.001]) and elderly patients (≥60 years, adjusted OR: 1.30 [1.24–1.36, *p*<0.001]) (Table 3 and Fig. 2B). Although a moderate increase was observed for children and adolescent (1–19 years, OR: 1.10 [95% CI 0.96–1.27, *p* = 0.177]), this rise was not statistically significant.

There were no prominent differences in mean VREFm proportions between female and male gender for the study period 2012–2018 (13.7% [95%CI 11.7–15.8%] and 12.9% [95%CI 11.4–14.6%], respectively) and in 2018 (19.8% [95% CI 17.1–22.9%] and 18.7% [95%CI 16.4–21.2%], respectively). However, multivariable logistic regression analyses showed an odds ratio of 0.91 (95% CI 0.84–1.0, *p* = 0.041) for vancomycin resistance in male compared to the female patients (Table 2). As shown in Table 3, the increase in VREFm proportions was observed in both female and male patients (OR: 1.23 [95% CI 1.13–1.36, *p*<0.001] and 1.27 [95% CI 1.22–1.33, *p*<0.001], respectively).

### Hospital unit type

**Figure 3.**
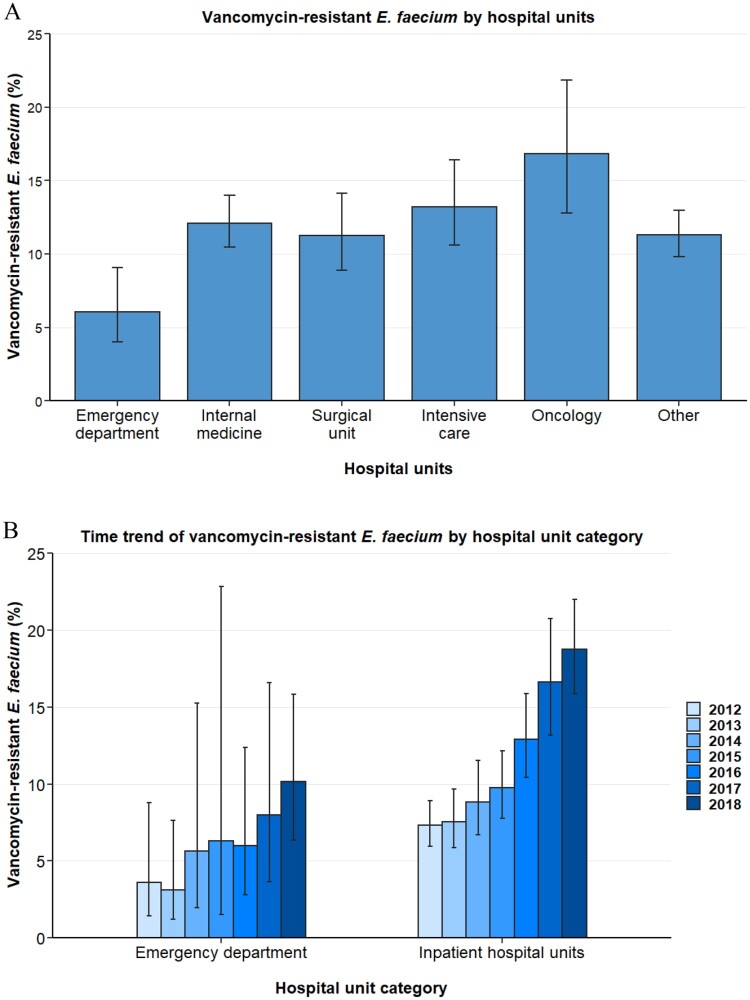
Vancomycin-resistant *E. faecium* from blood isolates stratified into hospital units. (A) Vancomycin-resistant *Enterococcus faecium* stratified into hospital units, expressed as population-weighted mean proportions (%) among all *Enterococcus faecium* blood isolates, with corresponding 95% confidence intervals. (B) Time trends of vancomycin-resistant *Enterococcus faecium* in blood isolates from the emgercency department and inpatient hospital units (internal medicine, surgical units, intensive care unit, onocology and other hospital units).

Analyses of *E. faecium* blood isolates per hospital unit type reveal substantial differences in vancomycin resistance proportions between isolates drawn in emergency departments and in inpatients hospital units such as internal medicine, ICU and surgical units (Fig. 3). While the population-weighted European mean VREFm proportion (2012–2018) was 6.1% (95%CI 4.0–9.1%) for the emergency department, considerably higher VREFm proportions were observed in internal medicine (12.1% [95% CI 10.5–14.0%]), intensive care (13.2% [95% CI 10.6–16.4%]) and surgical units (11.2% [95% CI 8.9–14.1%]) (Fig. 3). Since no pronounced differences were observed between internal medicine, intensive care, surgical, oncology and other hospital units, these units were aggregated into *inpatient hospital units* for logistic regression analyses. Univariable and multivariable regression analyses confirmed that the likelihood of VREFm in blood isolates from inpatient hospital units was markedly higher than in isolates from the emergency department (OR: 2.29 [95% CI 1.56–3.37, *p* < 0.001]) (Table 2).

Importantly, the rise of VREFm between 2012 and 2018 was seen in inpatient hospital units, where VREFm proportions rose from 7.3% (95% CI 6.0–8.9%) to 18.7% (95% CI 15.9–22.0%) (Fig. 3B). In line with this, multivariable regression analyses assessing the interaction between year and hospital units showed a statistically significant VREFm increase in inpatient hospital units (OR: 1.29 [95% CI 1.24–1.34, *p*<0.001]) (Table 3). Although an increase of VREFm proportions was found in emergency departments between 2012 (3.6% [95% CI 1.4–8.8%]) and 2018 (10.2% [95% CI 6.4–15.8%]) (Fig. 3B), the rise was not statistically significant in multivariable regression analysis (OR: 1.11 [95% CI 0.97–1.28, *p* = 0.121]) (Table 3).

### Co-resistance and cross-resistance

*E. faecium* blood isolates were evaluated for co- and cross-resistance with a range of antibiotics that are documented as alternative therapy options for vancomycin-resistant *E. faecium* infections, either as monotherapy or in combination with other drugs. We tested for co-resistance (amoxicillin, ampicillin, linezolid and gentamicin) and cross-resistance (teicoplanin) among vancomycin-sensitive and vancomycin-resistant *E. faecium* isolates (Table 4). The majority of *E. faecium* isolates that were resistant to vancomycin also exhibited high co-resistance to the penicillin antibiotics: ampicillin [99.4% (95% CI 99.2–100%)] and amoxicillin [99.3% (95%CI 98.2–100%)]. In comparison, vancomycin-sensitive *E. faecium* isolates (VSEF) also showed high resistance to ampicillin (88.9% [95% CI 88.1–90.0%]) and amoxicillin (84.8% [95% CI 82.5–87.0%]) but co-resistance proportions were lower than those from vancomycin-resistant *E. faecium* isolates. Considerable co-resistance among vancomycin-resistant and -sensitive *E. faecium* isolates was also found for gentamicin, a commonly used aminoglycoside (VREFm: 48.5% [95% CI 44.7–52.0%], VSEF: 43.4% [95% CI 41.0–46.0%]). Notably, co-resistance to linezolid, an alternative treatment option for VREFm infections, was very low among vancomycin-resistant *E. faecium* isolates (1.8% [95%CI 1.5–2.0%]). Cross-resistance to the glycopeptide antibiotic teicoplanin was very low among vancomycin-sensitive *E. faec*ium isolates (0.4% [95%CI 0.2–1.0%]) but high among vancomycin-resistant *E. faec*ium isolates (80.4% [95% CI 77.3–83.0%]) (Table 4).

**Table 4.** Co-resistance and Cross-resistance of vancomycin-resistant and -sensitive blood isolates to selected antibiotics.

		Vancomycin-resistant *E. faecium*	Vancomycin-sensitive *E. faecium*
*Co-resistance*			
	Ampicillin	99.4% (95% CI 99.2–100%)	88.9% (95% CI 88.1–90.0%)
	Amoxicillin	99.3% (95% CI 98.2–100%)	84.8% (95% CI 82.5–87.0%)
	Gentamicin	48.5% (95% CI 44.7–52.0%)	43.4% (95% CI 41.0–46.0%)
	Linezolid	1.8% (95% CI 1.5–2.0%)	1.13% (95% CI 1.0–1.3%)
*Cross-resistance*			
	Teicoplanin	80.4% (95% CI 77.3–83.0%)	0.4% (95% CI 0.2–1.0%)

### Vancomycin-resistant *E. faecalis*

**Additional Figure 2.**
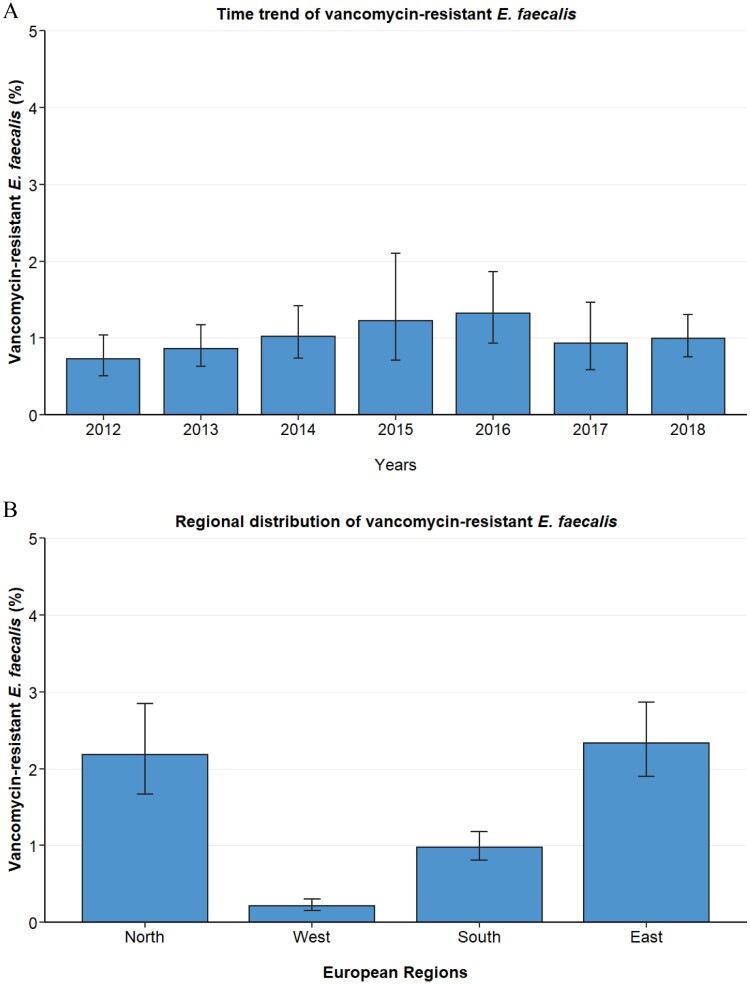
Vancomycin-resistant *E. faecalis* blood isolates in the EU/EAA. (A) Time trend of vancomycin-resistant *Enterococcus faecalis* in 30 countries of the European Union, Euroepan Economic Area and the United Kingdom, expressed as population-weighted mean proportions (%) among all *Enterococcus faecalis* blood isolates, with corresponding 95% confidence intervals. (B) Proportions of vancomycin-resistant *Enterococcus faecalis* in major regions of Europe.

We analysed proportions of vancomycin resistance among 103,112 *E. faec*alis isolates from patients with bloodstream infections (see Table 1 for baseline characteristics of the included *E. faecalis* blood isolates). During the whole study period, the European population-weighted mean proportion of vancomycin resistance in *E. faecalis* isolates was 1.1% (95% 0.9–1.4%). The proportion of vancomycin-resistant *E. faecalis* remained relatively stable between 2012 and 2018 (additional Figure 2A). Similar to *E. faecium*, proportions of vancomycin resistance among *E. faecalis* differed substantially between European regions. While in the North and East, proportions of 2.2% (95% CI 1.7–2.9%) and 2.3% (95% CI 1.9–2.9%) were observed, only 0.21% (95% CI 0.15–0.30%) and 1.0% (95% CI 0.81–1.2%) of all *E. faecalis* blood isolates exhibited vancomycin resistance in the West and South, respectively (additional Figure 2B).

## Discussion

Improved understanding of the interplay of factors that drive antimicrobial drug-resistant infections provides opportunities to address the associated clinical and public health burden on individuals, health systems, and society [26,27]. Therefore, we analysed isolates of *E. faecium* and *E. faecalis* collected from patients with bloodstream infections in hospitals across Europe between 2012 and 2018 using EARS-Net data.

Our analysis showed that there was a profound increase of vancomycin resistance in *E. faecium* blood isolates between 2012 (8.1%) and 2018 (19.0%) in EU/EEA countries (including the United Kingdom). Despite this increase, the 2018 mean VREFm proportion in Europe is still lower than current data reported from other parts of the world, such as the United States (66%) [28], Australia (47%) [7] and countries from the Eastern Mediterranean region such as Iran [29–31]. However, VREFm proportions reported in this study are significantly higher than those observed in Chinese hospitals, where VRE rates of lower than 2% were observed [32–34]. Similar to the situation in the EU/EEA, increasing VRE proportions have also been described in countries of the Eastern Mediterranean region [29]. Contrary to the trend in Europe, a large multicentre study showed that VREFm proportions in blood isolates reduced from 80.7% in 2010 to 66% in 2016 in the United States [28].

The rising trend in the EU/EEA countries is concerning since *E. faecium* infections are not only a major cause of nosocomial bloodstream infection but are also associated with a considerable disease burden. A recent study estimated that vancomycin-resistant enterococcus infections accounted for approximately 16,000 infections and 1000 attributable deaths in 2015 in EU/EEA countries [35]. In line with the rise of vancomycin resistance observed in our study, the study of Cassini et al. found that the number of vancomycin-resistant enterococci infections and attributable deaths almost doubled between 2007 and 2015 [35]. Compared to the situation in Europe, the burden of vancomycin-resistant enterococci is steadily decreasing in the Unites States, where the estimated number of cases in hospitalized patients declined from 85.000 in 2012–54,500 in 2017, most likely explained by increased infection control efforts and appropriate antibiotic use [13].

Importantly, our study shows that VREFm proportions in blood isolates increased in all EU/EEA regions and the majority of countries recorded a relative increase of VREFm rates. In addition, regional analyses showed that in 2018, VREFm was more pronounced (about two-fold higher) in the Northern and Eastern regions compared to the Southern and Western region of Europe. This is a contrast to the usual North/West - South/East gradient of antibiotic resistance for many pathogens observed in the EU/EEA including *Acinetobacter* spp., *Pseudomonas aeruginosa* and *Klebsiella pneumoniae* [9]. However, a considerable intra-regional heterogeneity was observed for the four major European regions at country level. For example, Ireland and the United Kingdom had considerably higher VREFm proportions than other northern countries, such as Norway and Sweden. Moreover, previous studies have shown that VREFm proportions can also significantly differ within individual countries as described for Germany, where a strong north–south disparity was observed [15]. The wide regional differences suggest that peculiar local factors might be driving the differences in vancomycin resistance among *E. faecium* isolates, such as VRE(Fm) diagnostics, infection control measures including active surveillance and varying antibiotic use. Local studies that incorporate demographic, treatment and clinical outcome data can delineate these unique factors that drive vancomycin resistance especially among hospitalized patients.

Our study found that adult and elderly patients with *E. faecium* bloodstream infections have a higher risk for VREFm than younger patients. Higher vancomycin resistance proportions in adults and elderly patients with enterococci infections have also been described in other studies [15,36]. In addition, similar age trends have been reported for other bacterial pathogens, including *Escherichia coli, Staphylococcus aureus, Streptococcus pneumoniae, Pseudomonas aeruginosa, Helicobacter pylori and Klebsiella pneumonia* [37–40]. As shown for enterococci, older patients are more likely to be colonized (and subsequently infected) with drug-resistant organisms due to more frequent exposure to antibiotics throughout their lives, thereby promoting the selection of drug-resistant bacteria [41]. Another possible reason is the more frequent exposure of older patients to long-term care facilities or other healthcare facilities, a known “reservoirs of resistance” due to poor infection control and prevalent use of broad-spectrum antibiotics [42,43]. In addition, the increasing trend in VREFm proportions was seen across all age groups except children and adolescents between 1 and 19 years. This may be explained by the lower frequency of hospital admission and/or lower consumption of antibiotics among children and adolescents in Europe compared to other age groups [44]. Increasing VREFm proportions in infants not neonates and elderly patients is of concern since ineffective antibiotic therapies are associated with increased mortality and morbidity in these vulnerable patient groups [45–47].

With respect to the origin of isolates, our study revealed that *E. faecium* isolates from inpatient hospital units, including ICUs and internal medicine, have a substantially higher proportion of vancomycin resistance compared to isolates from patients in the emergency department. A plausible explanation for this is the fact that patients treated in emergency departments most likely acquired the *E. faecium* infection in the community, rather than in the hospital. This interpretation is consistent with evidence from previous studies that reported a lower prevalence of vancomycin resistance among *E. faecium* isolates from emergency and outpatient hospital clinics compared to isolates from hospital inpatients [30,36,48]. In addition, we found that VREFm proportions significantly increased in inpatient hospital units over the years. This might be explained by increased use of invasive medical devices among inpatients, since the role of medical devices in the transmission of multidrug-resistant *E. faecium* infections is well documented [20]. Moreover, these inpatient hospital units are also characterized by the use of broad-spectrum antibiotics and patient populations that have serious pathologies and are more likely immunocompromised, which lowers their colonization resistance to known opportunistic pathogens like VREFm [49].

The very high cross-resistance of vancomycin-resistant *E. faecium* isolates to teicoplanin (80.4%) suggests that the *VanA* resistance gene is the main driver of glycopeptide resistance in *E. faecium* in the EU/EEA. Our finding of significant co-resistance of VREFm to ampicillin (>99%) and gentamicin (48.5%) underlines the progressively limited value of these antibiotics in the empirical management of VREFm infections, even when used in synergistic combinations [20,50]. However, it is encouraging that less than 2% of all VREFm blood isolates in this study exhibited co-resistance to linezolid, a last line drug used to treat vancomycin-resistant enterococci infections [51–53]. This is much lower than co-resistance proportions among vancomycin-resistant *E. faecium* enteric isolates in single centre studies in US (17.1%) and Italy (10.7%) [54,55]. A recent review of surveillance data by Bender et al. calls for caution since an increasing trend - albeit at low level – of linezolid resistance in VREFm, and enterococci in general, is emerging across Europe [56]. Such co-resistance presents additional challenges for patient treatment and infection control measures [50]. Therefore, there is a need to delineate the genomics of linezolid resistance determinants in *E. faecium* due to its increasing occurrence and the need for early implementation of adequate empiric VREFm treatment [54,57,58]. Molecular methods with high discriminatory power like wide genome sequencing will help to better understand the resistance mechanisms of emerging linezolid-vancomycin-resistant *E. faecium.* Such understanding is necessary to preserve its effectiveness – considering the diminishing antibiotic pipeline [59] – through stewardship activities and epidemiological surveillance of linezolid resistance across the EU/EEA countries.

In contrast to the results for *E. faecium,* we found that only 1.1% of *E. faecalis* blood isolates were vancomycin-resistant and no apparent increase was recorded between 2012 and 2018. In comparison, the proportion of vancomycin-resistant *E. faecalis* in blood isolates is 1.9–5.3% in the United States [28], whereas in China proportions are lower than 1% among isolates from different specimen material [32,34]. Our findings indicate the necessity to analyse vancomycin resistance patterns distinctively for *E. faecium* and *E. faecalis* in order fully understand the extent of the vancomycin resistance in enterococci infections. However, many epidemiological studies and surveillance programs do not differentiate enterococci to the species level. The proportion of vancomycin-resistant isolates in these studies is heavily influenced by the ratio of *E. faecium* and *E. faecalis*. Evidence from previous studies showed that the ratio of *E. faecium* and *E. faecalis* recorded in enterococci infection greatly differed among several individual studies [15,28,32,33].

### Strengths and limitations

This study included over 67,000 and 103,000 clinical blood isolates of *E. faecium* and *E. faecalis*, respectively, and is to our knowledge the largest and most comprehensive analysis of the vancomycin resistance profile of enterococci bloodstream infections in the EU/EEA. The analysed dataset is derived from routine clinical antimicrobial susceptibility data from national surveillance programs. The continuous collection of these AMR data allows for a longitudinal analysis; a very important indicator of trend. In addition, the regular external quality assessments of participating laboratories have demonstrated the validity of these AMR data [60]. While participating national laboratories and hospitals might not be fully representative for individual countries, over half of the countries reported a national coverage greater than 80% while population, hospital, and isolate sample representativeness was assessed as high in 25 countries [9]. Another limitation is the wide variation in population coverage among reporting countries. To minimize possible bias from differences in population size and isolate numbers from various countries, all statistical analyses used weighting based on the population sizes of the individual countries. Lastly, different sampling routine and admission characteristics (e.g. stay duration, bed space density) in different healthcare settings can result in biased estimates of VREFm proportions. However, the inclusion of only clinical bloodstream specimen limits the bias that may result from some of the inconsistencies in sampling, even though the frequency of blood sampling varies between hospitals and countries.

## Conclusion

This study demonstrated that vancomycin resistance in enterococci blood isolates is mainly reported for *E. faecium* isolates. The rising trend of vancomycin-resistant *E. faecium* is pervasive across the EU/EEA and particularly among hospitalized adult and elderly patients. These findings have implications for patient care and justify the need to analyse the available data more rapidly at country level and also identify specific regions with high VREFm within the countries. National and regional authorities should intensify efforts directed at diagnostic and antimicrobial stewardship for vancomycin and all last resort drugs for the control of nosocomial enterococci infections.

## Geolocation information

Europe
